# Effect of four neuraminidase inhibitors on influenza in Osaka, Japan: An eight‐year survey

**DOI:** 10.1002/jgf2.286

**Published:** 2019-10-31

**Authors:** Yoshihiro Tochino, Naoko Yoshii, Masashi Fujioka, Takashi Hamazaki, Tomoya Kawaguchi, Hiroshi Kakeya, Taichi Shuto

**Affiliations:** ^1^ Department of Medical Education and General Practice Osaka City University Graduate School of Medicine Osaka Japan; ^2^ Department of Respiratory Medicine Graduate School of Medicine Osaka City University Osaka Japan; ^3^ Department of Infection Control Science Osaka City University Graduate School of Medicine Osaka Japan; ^4^ Fujioka Pediatric Clinic Osaka Japan; ^5^ Department of Pediatrics Graduate School of Medicine Osaka City University Osaka Japan

**Keywords:** fever, influenza, neuraminidase inhibitor, questionnaire

## Abstract

**Background:**

After the A/H1N1 influenza pandemic in 2009, two new drugs against influenza, namely laninamivir and peramivir, were released in 2010 in Japan. We investigated prescription trends of four neuraminidase inhibitors (NAIs), which are laninamivir, peramivir, oseltamivir, and zanamivir, and assessed clinical data related to influenza for 8 years.

**Methods:**

Patients living in Osaka Prefecture and diagnosed with influenza responded to a postcard questionnaire that collected data regarding their demographic characteristics, symptoms including fever, prescribed drugs, and influenza type.

**Results:**

Laninamivir was most prescribed to patients aged ≥ 10 years, and oseltamivir was most prescribed to patients aged < 10 years. All four NAIs had similar effects on influenza. Patients with type A influenza experienced fever alleviation earlier than those with type B influenza. Older patients tended to have lower fever. Most seasons had similar results.

**Conclusions:**

Our descriptive epidemiologic study revealed the status of patients with influenza and their medication use.

## Graphical abstract

We showed the data of eight seasons and report prescription trends of four neuraminidase inhibitors (NAIs) and time course of fever in patients after taking these inhibitors. Doctors, especially general physicians, should consider how to choose anti‐influenza drugs. This study based on our 8‐year survey should be helpful to them when deciding which NAIs should be chosen in clinical settings and for which patients.

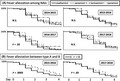

## 1 INTRODUCTION

After the A/H1N1 influenza pandemic in 2009, two new drugs against influenza, namely laninamivir and peramivir, were released in 2010 in Japan. Particularly, laninamivir was released only in Japan. Four neuraminidase inhibitors (NAIs) are typically used after rapid influenza diagnostic tests: oseltamivir (orally administered twice daily for 5 days), zanamivir (inhaled twice daily for 5 days), laninamivir (inhaled just once on the first day), and peramivir (intravenously administered once on the first day or once daily for a maximum of 3 days).

Influenza is one of the most common viral infections affecting global communities annually. During influenza season, doctors typically perform rapid influenza diagnostic tests for patients with sudden‐onset high fever. If tested positive, NAIs can be prescribed to patients, even to those with no risk factors. Meta‐analyses of 2009 influenza pandemic showed that early NAI treatment versus late was associated with a significant reduction in mortality.^1^ Sugaya N. et al also showed early treatment with NAIs led to very low mortality rate in children during the 2009 influenza pandemic in Japan.^2^

Patients diagnosed with influenza are prescribed NAIs in outpatient clinics, after which they rarely revisit the hospital or clinic. Therefore, it is difficult for doctors to know the treatment outcomes of these patients. During the winter season, doctors are too busy to investigate detailed treatment outcomes owing to the volume of patients with viral infections, cardiovascular diseases, or respiratory diseases. Therefore, we conducted a less time‐intensive self‐reported survey using a postcard questionnaire. This method poses a lower burden for doctors and for patients with influenza previously treated with NAIs.^3^, ^4^, ^5^, ^6^

To investigate the status of patients with influenza and their medication use, we distributed postcard survey questionnaires to patients in Osaka Prefecture from the 2010‐2011 winter season. During 2010‐2011 season, laninamivir appeared to alleviate fever faster, with fewer adverse events.^3^ During 2011‐2012 season, laninamivir was prescribed more often than during the previous season and the four NAIs had similar fever‐alleviating effects.^4^ Furthermore, we had reported that during the 2012‐2013 and 2013‐2014 seasons, fever in patients with type A influenza was alleviated earlier than those with type B influenza.^5^, ^6^

This descriptive epidemiologic study was performed to reveal three points. (a) Difference of each NAI's effects: We report prescription trends of four NAIs and time course of fever in patients after taking these inhibitors; (b) difference between influenza type; and (c) difference of fever in patients of influenza by ages. We present data of eight seasons.

## 2 MATERIALS AND METHODS

**Figure 1:**
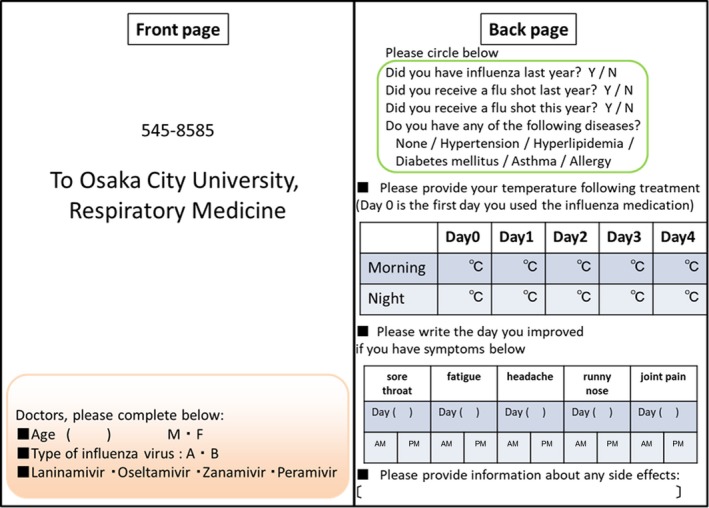
Sample postcard from the 2012‐2013 season. Front side information was completed by doctors, and back side information was completed (and returned via post) by patients

This study is observational, cross‐sectional study of influenza for eight years. This study was conducted in 72 hospitals or clinics in Osaka Prefecture, and some institutions were changed by the season. In the first year of this survey, we recruited institutions which are recommended by department of respiratory medicine, pediatric medicine, and otolaryngology of Osaka City University. As of hospitals, specialists of internal medicine, pediatric medicine, and otolaryngology were in charge of this survey. Each clinic was in charge of 10‐30 postcards, depending on their institutional capacity. Overall, 833‐1050 postcards were prepared for distribution by clinics. Inclusion criteria were that patients were diagnosed using rapid influenza diagnostic tests, and NAIs were prescribed between December and April the next year in every year for 8 years. Doctors delivered postcards by convenient sampling. After informed consent was obtained, doctors entered information on the patient's age, sex, influenza type (A or B), and drug details on the front of the postcards and provided them to the patients. After returning home, patients answered questions regarding their highest temperature (twice daily), flu vaccination status, and other symptoms. (Figure 1) Then, the postcards were returned to Osaka City University.

Information reported during the 2011‐2012 season included their highest temperature (recorded twice daily, in the morning and in the evening) and the presence or absence of sore throat, fatigue, headache, runny nose, and joint pain. A sample postcard from the 2012‐2013 season is presented in Figure 1. Highest fevers were asked for 8 years, but other questions have been changed partly by reviewing results and questions every year. Cases with missing data on age, sex, influenza type, or prescribed NAIs were excluded.

We defined the duration of fever as the time from NAI administration until the patients became afebrile. Fever reduction was defined as temperatures < 37.5°C in patients aged < 10 years or < 37.0°C in those aged ≥ 10 years, as previously described.^7^, ^8^ This study was approved by the Ethics Committee of Osaka City University (Approval No. 2465, 3000).

### 2.1 Statistical analysis

Statistical analyses were performed with JMP (version 10; SAS Institute, Inc). Mann‐Whitney test was performed to compare the proportion of NAIs between two age groups of type A influenza. A log‐rank test was used to compare about fever alleviation among NAIs or influenza type. A Fisher's exact test was used to compare the proportion of highest fever among age groups. For all statistical analysis, *P* values of <.05 were considered significant.

## 3 RESULTS

Characteristics of this survey are displayed in Table 1. Gender was mostly even. Approximately 15% of patients had other diseases such as hypertension, hyperlipidemia, diabetes mellitus, and asthma. Most patients were administered NAIs within 48 hours and approximately under 10% of patients were still not afebrile at 5 days after taking NAIs. The return rate was also displayed in Table 1. The return rate was approximately 40% each year.

**Table 1:** Characteristics and clinical feature of patients for eight years

	Gender	Age		Type	Other diseases,%	NAIs over 48 h, %	Non afebrile, %	Return rate, %
	Male/Female	9 and under	10 and over	A/B				
2010‐11	126/121	130	117	179/68	13.0 (32/247)	N/A	4.9 (12/247)	25.8 (249/963)
2011‐12	158/155	163	150	274/39	11.2 (35/313)	N/A	3.2 (10/313)	39.7(330/833)
2012‐13	116/125	116	125	223/18	10.8 (26/241)	N/A	4.6 (18/241)	30.5 (263/863)
2013‐14	159/148	150	157	232/75	14 (43/307)	N/A	9.1 (28/307)	31(326/1050)
2014‐15	181/165	121	225	337/9	19.4 (67/346)	N/A	6.6 (23/346)	35.9 (359/1000)
2015‐16	150/182	134	198	187/145	16.6 (55/332)	2.1 (7/332)	12 (40/332)	40 (360/900)
2016‐17	164/177	113	228	340/1	19.7 (67/341)	0.3 (1/341)	4.5 (12/341)	39.1 (352/900)
2017‐18	131/156	132	155	147/140	18.9 (54/287)	1.4 (4/287)	7.3 (21/287)	34.2 (308/900)

### 3.1 Laninamivir was most prescribed to patients aged ≥ 10 years, and oseltamivir was most prescribed to those aged < 10 years

**Figure 2:**
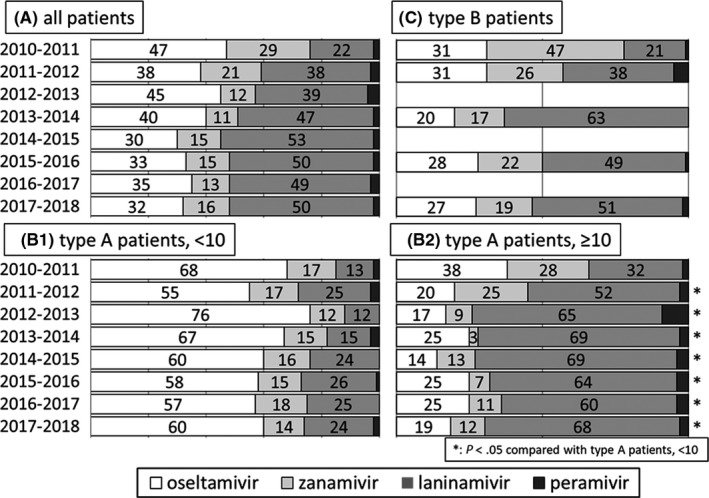
Proportion of the four prescribed neuraminidase inhibitors for each of the eight seasons. A, All patients. B‐1,2, Patients with type A influenza further divided into two groups by age. There were significant differences in proportion of NAIs. C, Patients with type B influenza

Figure 2 presents the proportion of patients taking each NAI during each of the eight seasons. Laninamivir was the most‐prescribed NAI in all patients. Peramivir was prescribed to only a small percentage of patients each year (Figure 2A). Four years after the release of laninamivir and peramivir, the proportion of patients taking each NAI stabilized: laninamivir, 49%‐53%; oseltamivir, 30%‐35%; zanamivir, 13%‐16%; and peramivir, 2%‐3%.

Among patients diagnosed with type A influenza, laninamivir was prescribed to significantly older patients (typically aged ≥ 10 years), whereas oseltamivir was prescribed to significantly younger patients (aged < 10 years) since the 2011‐2012 season (*P* < .05) (Figure 2 B‐1, 2). As seen in Figure 2C, only around 20 patients were diagnosed with type B influenza during each season. Zanamivir was prescribed to approximately 20% of patients with type B influenza. This NAI was preferred for use in patients with type B influenza compared to those with type A.

### 3.2 All NAIs had similar effects on influenza

**Figure 3:**
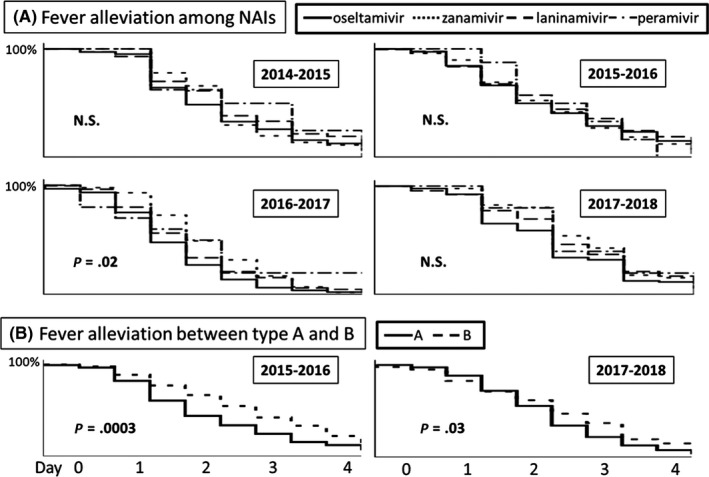
Comparison of fever alleviation. A, Fever alleviation associated with the four neuraminidase inhibitors from the 2014‐2015 to 2017‐2018 season. B, Comparison of fever alleviation in patients with type A and B influenza during the 2015‐2016 and 2017‐2018 season

The time until fever alleviation did not differ according to the NAI used except the 2016‐2017 season. Figure 3A shows representative results from the 2014‐2015 season to 2017‐2018 season. The data before 2013‐2014 season were already reported in previous papers.^3^, ^4^, ^5^, ^6^ Most seasons had the same results about the effects of NAIs for alleviation of fever.

### 3.3 Patients with type A influenza achieved fever alleviation earlier than those with type B influenza

The time until fever alleviation was compared between patients with type A influenza and those with type B influenza during the 2015‐2016 season and 2017‐2018 season (Figure 3B). We did not show the data of 2014‐2015 and 2016‐2017 season because of small number of influenza type B patients. The data before 2013‐2014 season were already reported in previous papers, and they were mostly the same results.^3^, ^4^, ^5^, ^6^ Patients with type A virus achieved fever alleviation significantly earlier.

### 3.4 Older patients had significantly lower fever

**Figure 4:**
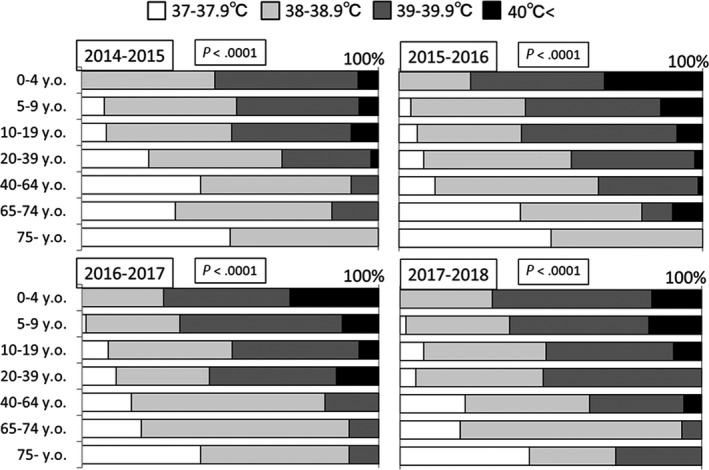
Proportion of patients with highest fever, by age group. Note that older patients significantly experience lower fevers

Figure 4 shows the proportion of patients with the highest temperatures according to age group from 2014‐2015 to 2017‐2018 season. We did not show the data before 2013‐2014 season because they were already published.^3^, ^4^, ^5^, ^6^ The number of patients with fever ≥39°C gradually decreased and that of patients with fever <38°C gradually increased with age (*P* < .0001). Thus, older patients with influenza may not uniformly exhibit high fever.

## 4 DISCUSSION

This 8‐year survey showed the prescription trends of four NAIs during each season. Laninamivir is used only in Japan and was the most‐prescribed NAI. Patients of similar ages and influenza type had similar proportions of prescribed NAIs during each season. Presumably, the NAIs were chosen considering the patients' ages, drug compliance, and number of doses. These data can be a useful reference for general physicians and clinicians of internal medicine, otolaryngology, and pediatrics.

There was no difference in the time to alleviate fever among the four NAIs prescribed to patients with influenza. Proper prescription of anti‐influenza drugs requires a physician to understand the characteristics of each NAI, which is explained below. Laninamivir is administered via a one‐time inhalation and may, therefore, be associated with a high rate of compliance. However, small children, particularly those aged < 3 years, have difficulty inhaling laninamivir.^7^ Peramivir should be infused via an intravenous drip for more than 15 minutes. Therefore, peramivir was prescribed to few patients to prevent the spread of influenza in clinics or hospitals during long hospital stays. Oseltamivir and zanamivir were administered for longer durations than laninamivir and peramivir. Importantly, patients need to complete five full days of these drugs and not discontinue early or when they begin to feel better. The National Institute of Infectious Diseases of Japan has reported that anti‐influenza drug resistance was rarely observed during 8 years,^9^ potentially leading to similar effects of the four NAIs. Anti‐influenza drugs should be chosen based on the individual patient's condition and drug characteristics.

Among patients with type A influenza, fever was alleviated earlier than in those with type B influenza. This trend held true almost every year, and older patients had lower fevers. Many studies have reported that patients with type A influenza experienced better efficacy with NAIs than those with type B influenza,^10^, ^11^ and these findings are in agreement with the results of this study. Importantly, we obtained nearly identical results each year. In general, adults aged ≥ 65 years exhibited attenuated febrile responses. This might reflect altered thermoregulatory responses or lower baseline core body temperatures.^12^, ^13^, ^14^, ^15^, ^16^, ^17^ Our results are concurrent with those of previous studies; moreover, to the best of our knowledge, this may be the first report to confirm that maximum fever gradually lowered according to age in patients with influenza. These results indicate that even if elderly patients do not have high fever, for example under 38°C, we should consider a possibility of influenza infection.

Although this postcard survey was relatively easy to conduct and produced results with a low burden for doctors and patients, three limitations exist. The first limitation is bias of doctors and patients. Doctors' biases involve a prescription bias and a selection bias. Doctors deciding which NAI to prescribe could produce a prescription bias, which is influenced by prescribers' belief, drug access, or other social factors. We had previously reported that there were no significant differences in sex, age distribution, or choice of prescribed drugs between our survey and the Japan Physicians Association report, which is one of the most reliable investigations of influenza in Japan.^3^, ^4^, ^5^, ^6^, ^10^ This study used the convenience selection which led to a selection bias of doctors. This survey only included patients who visited clinics and underwent rapid influenza diagnostic tests, which also led to a selection bias, too. Patients' biases involve a reporting bias and a recall bias, which lead to an information bias. This type of study may include some inaccurate data of patients. The second limitation was the postcard return rate. Our observed return rate was approximately 40%, which was not very high. Therefore, the sample may not be representative of all patients with influenza. Only patients who could measure fever twice a day might return postcards. These results may reflect that all patients did not measure fever twice a day. The Ministry of Health, Labour and Welfare had provided the number of prescriptions of NAIs in 2012‐2013 based on data furnished by pharmaceutical companies.^18^ These data showed the proportions of prescribed NAIs to be 45% 14%, 39%, and 2% for oseltamivir, zanamivir, laninamivir, and peramivir, respectively. These proportions are similar to our findings: 45% oseltamivir, 12% zanamivir, 39% laninamivir, and 4% peramivir. Therefore, our survey population might be representative supporting the reliability of our data. The third limitation is not to show a comparison between patients with NAIs and without NAIs. It is not possible to verify whether NAI is effective compared to nonuse.

It is very important for them to know the current status of the four NAIs. This study supported NAIs had mostly the same effect and better to alleviate fever in type A influenza patients than type B, or elderly patients did not have high fever in influenza infection.

In conclusion, our descriptive epidemiologic study about influenza patients for eight years revealed the status on patients of influenza and their medication use.

## CONFLICT OF INTEREST

Authors declare no conflict of interest for this article.
